# Correct Outcome Prognostication via Sonographic Volumetry in Supratentorial Intracerebral Hemorrhage

**DOI:** 10.3389/fneur.2019.00492

**Published:** 2019-05-08

**Authors:** Wolf-Dirk Niesen, Axel Schlaeger, Juergen Bardutzky, Hannah Fuhrer

**Affiliations:** ^1^Department of Neurology, Medical Center - University of Freiburg, Freiburg, Germany; ^2^Department of Neurology, Medical Center Esslingen, Esslingen, Germany

**Keywords:** stroke, intracerebral hemorrhage, sonography, transcranial ultrasound, volumetry

## Abstract

**Introduction:** The intracerebral hemorrhage (ICH)-score is used for estimation of patients' prognosis. The hemorrhage volume calculated from computed tomography (CT) contributes as one main factor. Several studies have proven that dimensions of an ICH may be displayed sufficiently by transcranial sonography (TCS). Yet, the adequacy of ICH-volumetry via TCS in calculating the ICH-score and its use as prognostic tool has not been studied.

**Methods:** Forty consecutive patients with supratentorial ICH diagnosed via CT were included in this prospective observational pilot study. 45 examination-series via CT and TCS were done in order to perform an ICH-volumetry and calculate the ICH-score. Volume was calculated using the ABC/2 estimation. Results of both imaging techniques were compared regarding quantification of ICH- volume and correct prognostication. A modified Rankin Scale (mRS)-score of 0–3 points was valued as good outcome.

**Results:** The imaging techniques did not show a difference in volumetry (*p* = 0.794) and TCS derived hemorrhage volume correlated significantly with ICH-volume measured on CT-scans. Calculated ICH-scores also did not differ (*p* = 0.323). Patients with an ICH-score larger than 2 points were predicted to experience a poor outcome at discharge with mRS 4–6 points, and the prognostication of the outcome was correct. Patients with a good outcome showed a smaller ICH-volume (11.2 ± 9.1ml) than patients with a poor outcome (38.2 ± 41.2 ml; *p* = 0.002).

**Conclusion:** Volumetry in supratentorial ICH via TCS is feasible and the prognostication with the ICH-score based on its results is comparable to CT-imaging and sufficient.

## Introduction

Intracerebral hemorrhages (ICH) account for 10–15% of all strokes and are associated with a poor prognosis. Although a relevant portion of patients receives maximal care, the overall mortality rate reaches up to 45% (1). Early prognostication is crucial in order to guide treatment measures. Several ICH-scoring systems have been developed to predict patients' outcome focusing on mortality. A widely used tool is the “ICH-score.” It comprises age, the Glasgow Coma Scale-Score, location of ICH (infratentorial), intraventricular hemorrhage and ICH-volume as independent prognostic factors associated with the 30-day outcome (2, 3). The ICH-volume is calculated using a simple volumetry based on the formula ABC/2 on CT-scans (4). A hemorrhage-volume greater than 30 ml is strongly associated with a poor outcome (2–4) and may be the most relevant factor to determine treatment (5).

Cerebral structures and pathologies can be visualized via transcranial gray-scale sonography (TCS) with a high sensitivity and specificity comparable to CT-scans. TCS also allows monitoring of ICHs and their resorption course (6). So far, it has not been studied if hemorrhage volume can be depicted accurately via TCS and if the ICH-score based on TCS-data is comparable to results using CT-scans in regard to patients' outcome.

## Methods

### Study Design

Forty consecutive patients with supratentorial ICH who were admitted to the Neurological Intensive Care Unit (NICU) of the University Medical Center in Freiburg were included into this prospective observational pilot study. ICH was diagnosed via CT-scans on admission by a neuroradiologist. The ICH-score was calculated based on clinical and imaging data as described previously (2, 3). Furthermore, we collected modified Rankin Scores (mRS) at discharge, and defined a mRS with 0–3 points as a good and 4 to 6 as a poor outcome. Additionally, a 30 day-mortality rate was estimated using the ICH-Score with ICH-volumetry based on CT and on TCS, respectively. This study was carried out in accordance with the recommendations of the local ethic committee of Freiburg and the Declaration of Helsinki. The local ethic committee of Freiburg approved the protocol (EK 28/10). As this is an observational study, the board waived the need for written patients' consent.

### Transcranial Sonography

**Figure 1:**
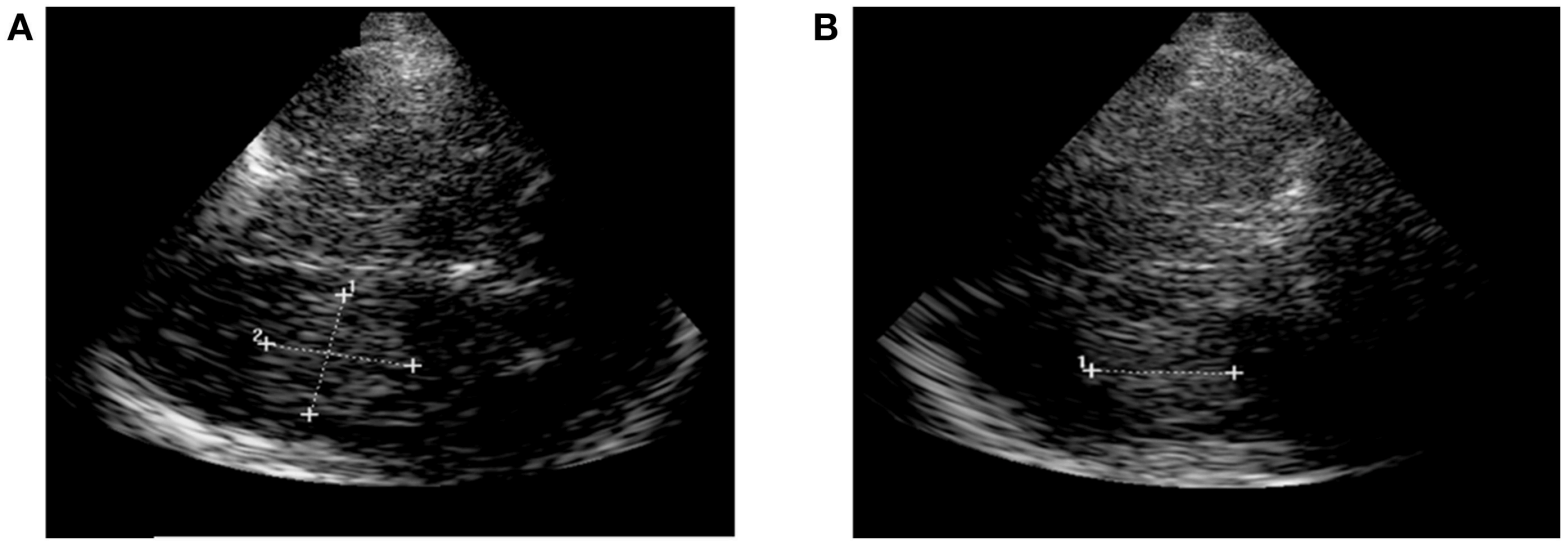
Sonographic ICH-volumetry. **(A)** Hemorrhage expansion measured in the axial plane (length and width of the ICH). **(B)** Hemorhage expansion measured in the coronal plane (height of the ICH). ICH, intracerebral hemorrhage.

TCS was done by experienced neurologists on admission. It was performed using a GE Logique 7 expert ultrasound system (GE Healthcare, USA) with a 2.0–2.5 MHz-transducer on the transtemporal approach in a meato-orbital plane. Visualization of hyperechogenic blood was performed progressing from the contralateral side of the lesion to a scanning-depth of 16 cm and tilting the probe to the maximum extent of the hemorrhage. So, first its length (A) was measured, followed by measuring the width (B) in a 90 degree-angle. Third, the dimension of the height (C) of the ICH was measured in the coronal sectional plane. ICH-volume was calculated by multiplying length (A), width (B) and height (C) divided by 2 (Figure 1) (4, 6). Volumetry was performed with examiner being blinded for CT-data.

### CT

Localization of ICH was described, and ICH-volumetry done on CT-scans with a slice-thickness of 5 mm analogously to TCS with ABC/2 by an experienced stroke neurologist blinded for TCS-data.

### Statistics

After testing for normal distribution clinical, TCS- and CT-data was compared descriptively and correlated via non-parametric statistics. TCS and CT derived ICH-scores were compared using non-parametric testing. Bland-Altman-analyses of TCS- and CT-volumetry were calculated; a maximum of a 10%-variance of volumes measured in comparison to CT would confirm validity of TCS (7). The first analysis was performed comparing all volumetric data. A second Bland-Altman-analysis was done in patients showing an ICH-volume on CT or TCS between 25 and 35 ml as an ICH-volume of 30 ml is the cut-off value for allocating one point in calculation of the ICH-score and differences in TCS- and CT-volumetry could lead to a score-difference. We further analyzed imaging-data in regards to functional outcome and mortality, and assessed whether prognostication on admission was correct in correlation to the outcome at discharge.

## Results

### Patient Characteristics

Forty patients diagnosed with ICH were included in this study. Patient characteristics are summarized in Table 1. In total, 45 TCS- and CT-examinations were performed. Median mRS at discharge was 4 points (interquartile range, IQR, 2-5). 5 (12.5%) patients died during the hospital stay. 14 patients were discharged with a good outcome (mRS-score 0–3).

**Table 1:** Patients' characteristics.

	***n* = 40**
Mean age, years (*SD*)	66.3 (11.4)
Male, *n* (%)	26 (65.0)
Mean GCS on admission, score (*SD*)	12 (2.8)
ICH location, *n* (%)
Deep ganglionic	21 (52.2)
Lobar	7 (17.5)
Infratentorial	3 (7.5)
Atypical	9 (22.5)
Additional intraventricular bleeding, *n* (%)	13 (32.5)

*ICH, intracerebral hemorrhage; n, number; SD, standard deviation*.

**Figure 2:**
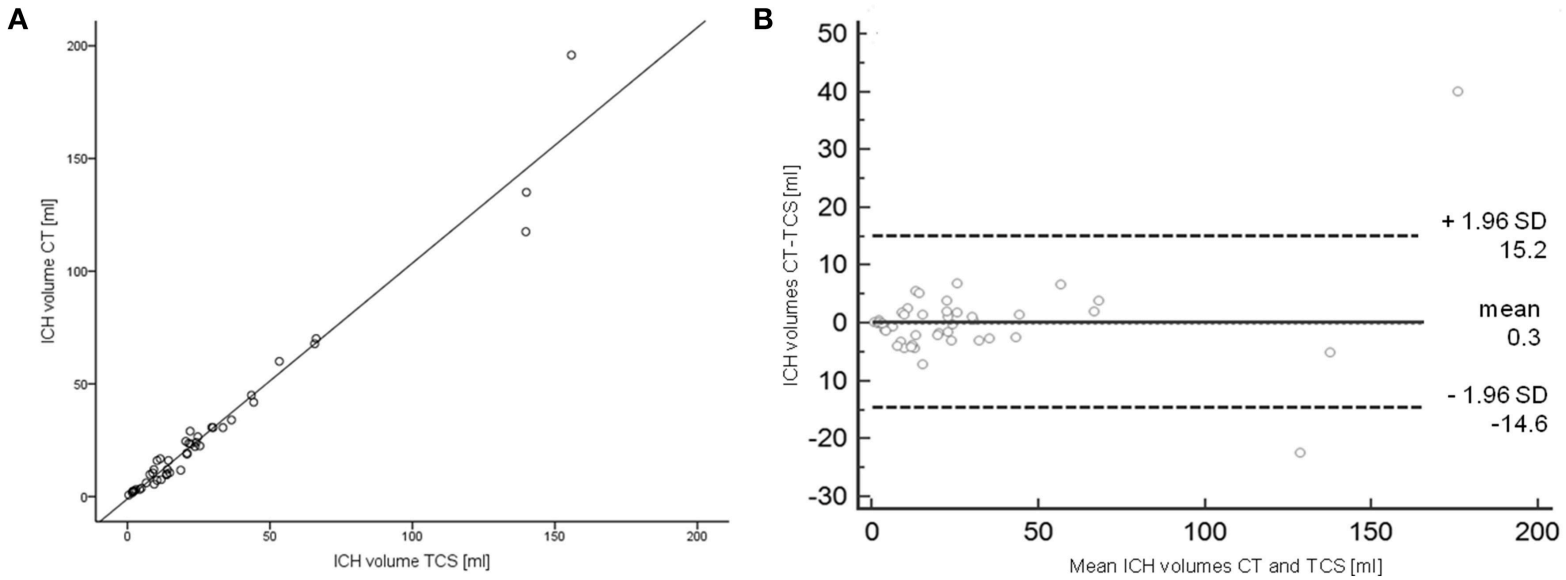
Correlation and Bland-Altman-analysis of TCS- and CT-data. **(A)** Linear correlation of hemorrhage-volumetry measured via CT and TCS. **(B)** Bland-Altman-analysis comparing CT- and TCS-volumetry. CT, computed tomography; TCS, transcranial sonography.

Median ICH-volumes measured with 16.8 ml (IQR 7.5–30.75 ml) on CT-scans and 18.7 ml (9.3–30.0) on TCS did not differ (*p* = 0.748). Volumes showed a strong linear correlation between TCS- and CT- ICH volumes (*r* = 0.981, *p* < 0.0001; Figure 2). ICH-resorption was monitored in 4 patients, also in linear correlation to CT-data. The Bland-Altman-analysis showed a bias of calculated volumes of both techniques of −1.88ml (6.7%). In six patients ICH-volumes on CT or TCS were in the range between 25 and 35 ml. Bland-Altman-analysis showed a bias of −2.63 ml (9.7%) in those cases. There was one patient in whom the ICH-volume was calculated with 29.6 ml on TCS, and 30.7 ml on CT. In that single case the ICH-score would differ by one point depending on the imaging-modality the calculation is based on.

Calculating the ICH-score with the CT-data revealed a median score of 1.0 (IQR 1.0–2.0) which did not differ from the TCS-ICH-score with a median of 1.0 (1.0–2.0; *p* = 0.323; correlation *r* = 0.988, *p* < 0.0001). Patients with an ICH-score > 2 were predicted to experience a poor outcome (mRS 4–6) at discharge. The predicted outcome did not differ from the actual outcome at discharge [χ^2^(2) = 2.577, *p* = 0.276].

In order to assess how TCS-derived volumetry data correlates with patients' outcome, patients were grouped according to their mRS-score at discharge. Fourteen patients had a good outcome (mRS 0–3) and 26 had a poor outcome (mRS 4–6).

**Figure 3:**
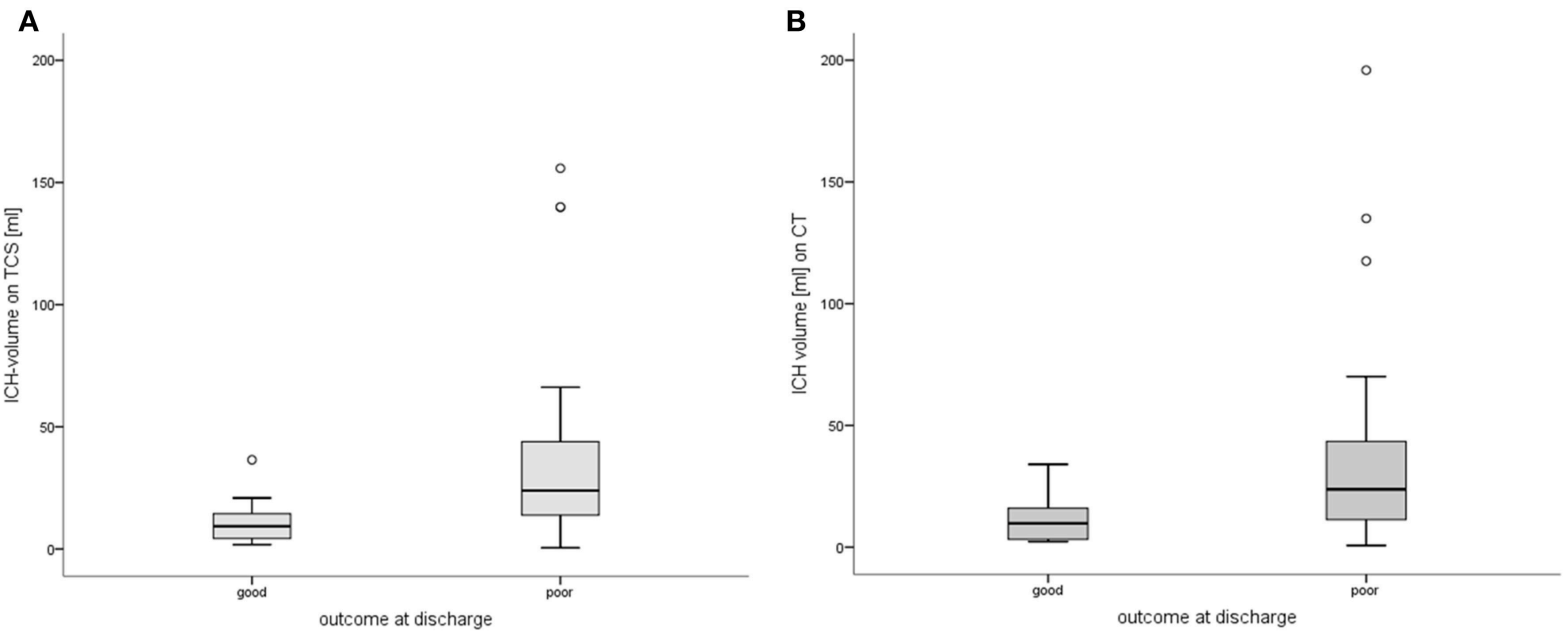
Volumes of intracerebral hemorrhages in patients with good vs. poor outcome. **(A)** Comparing patients with good vs. poor outcome based on TCS-volumetry (*p* = 0.002). **(B)** Comparing patients with good vs. poor outcome based on CT-volumetry (*p* = 0.013). Significant differences of ICH-volumetry in patients with a good (mRS 0-3) vs. poor (mRS 4-6) outcome at discharge. ° indicating outliers with discordant volumetric values. CT, computed tomography; ICH, intracerebral hemorrhage; mRS, modified Rankin Scale; TCS, transcranial sonography.

Patients with a good outcome had significantly smaller ICH-volumes based on TCS-examination compared to patients with a poor outcome [good: mean volumes 11.2 (±9.1) ml vs. poor: 38.2 (±41.2) ml; *p* = 0.002; Figure 3]. Comparable results could be found for volumetry based on CT-data, showing a larger ICH-volume in patients with poor outcome (*p* = 0.013; Figure 3). Furthermore, ICH-volumes differed significantly between single mRS-levels of 0 to 6 [χ^2^(6) = 13.491, *p* = 0.036]. We could not detect a difference of ICH-volumes in the 35 patients surviving the hospital stay and the 5 patients who deceased (*p* = 0.277).

## Discussion

The results of this prospective pilot study show that TCS-volumetry using the GE Logique 7 expert ultrasound system in supratentorial ICH is highly comparable to CT-data and therefore yields the same ICH-scores. ICH-volumes differed regarding outcome reflected by mRS-levels at discharge, and prognostication regarding a good or poor outcome was correct based on TCS volume-measurements and the ICH-score calculated from it.

Among the existing prognostic scores, the ICH-score is the most widely used in clinical practice. The ICH-score is a simple prognostic-tool. It comprises the GCS-score defining the severity of clinical symptoms, additionally modified by age, ICH-volume and –location (2, 3). A 30 day-prognosis on functional outcome can be made based on its results; we confirmed the correct prediction of short-term outcome at discharge. We were not able to show a difference in ICH-volumes regarding in house-mortality. Others, however, were able to demonstrate that the ICH-score can be used to predict a 30 day-mortality (2, 8).

It has been suggested to use surgical methods based on the ICH-score to reduce mortality in ICH-volumes greater than 30ml and GCS-scores below 12. ICH-Volume seems to be the strongest factor influencing patients' outcome (5, 9). The usage of the ABC/2-volumetry is comparable to CT-based planimetry but the method is limited in irregularly shaped or multinodular hematomas (10, 11).

We were able to examine all patients with TCS. Difference between TCS and CT-volumetry was very low in all patients. It is often pointed out that this technique is limited by insufficient transtemporal bone window (in 16–36% of the European population) (12). In general, small hemorrhages in the frontal or parasagittal region cannot be adequately assessed via TCS as the spatial resolution in these areas is limited (13). But all other areas can be depicted sufficiently. Despite these limitations we believe that TCS may not be used as primary diagnostic measure but is an excellent tool to assess ICH-expansion in a cost- and time-efficient way. Further, TCS may become especially handy in critically ill patients or patients needing repeated imaging. Side effects of transportation and x-ray-exposure could be reduced in those patients using TCS as a bedside-technique; it may then be relevant to evaluate their prognosis. We were able to use TCS as follow-up in 4 patients, again with high correlation to CT-based volumetry.

We conclude that calculating ICH-volume via TCS is feasible and that prognostication with the ICH-score based on its results is comparable to using CT-imaging.

## Ethics Statement

This study was carried out in accordance with the recommendations of the local ethic committee of Freiburg and the Declaration of Helsinki. The local ethic committee of Freiburg approved the protocol (EK 28/10). As this is an observational study, the board waived the need for written patients' consent.

## Author Contributions

HF did the literature research, performed the statistics and wrote the first draft of the manuscript. W-DN contributed conception. W-DN and JB designed the study. W-DN, AS, and JB gathered clinical, sonographic and radiological data. W-DN, JB, and AS contributed sections to the manuscript. All authors contributed to manuscript revision, read and approved the submitted version.

### Conflict of Interest Statement

AS received travel funding from Ipsen and speaking honoraria from Bayer Health Care. JB received speaking honoraria from Bayer Health Care, Boehringer Ingelheim, Daiichi Sankyo and Pfizer. The remaining authors declare that the research was conducted in the absence of any commercial or financial relationships that could be construed as a potential conflict of interest.
